# A meta-analysis of multiple stressors on seagrasses in the context of marine spatial cumulative impacts assessment

**DOI:** 10.1038/s41598-020-68801-w

**Published:** 2020-07-20

**Authors:** Jackson Stockbridge, Alice R. Jones, Bronwyn M. Gillanders

**Affiliations:** Southern Seas Ecology Laboratories and Environment Institute, School of Biological Sciences, University of Adelaide, Darling Building DX 650 418, Adelaide, SA 5005 Australia

**Keywords:** Marine biology, Environmental impact, Conservation biology

## Abstract

Humans are placing more strain on the world’s oceans than ever before. Furthermore, marine ecosystems are seldom subjected to single stressors, rather they are frequently exposed to multiple, concurrent stressors. When the combined effect of these stressors is calculated and mapped through cumulative impact assessments, it is often assumed that the effects are additive. However, there is increasing evidence that different combinations of stressors can have non-additive impacts, potentially leading to synergistic and unpredictable impacts on ecosystems. Accurately predicting how stressors interact is important in conservation, as removal of certain stressors could provide a greater benefit, or be more detrimental than would be predicted by an additive model. Here, we conduct a meta-analysis to assess the prevalence of additive, synergistic, and antagonistic stressor interaction effects using seagrasses as case study ecosystems. We found that additive interactions were the most commonly reported in seagrass studies. Synergistic and antagonistic interactions were also common, but there was no clear way of predicting where these non-additive interactions occurred. More studies which synthesise the results of stressor interactions are needed to be able to generalise interactions across ecosystem types, which can then be used to improve models for assessing cumulative impacts.

## Introduction

Humans rely on ocean ecosystem services and resources, and our growing population means demand for these services is rising^1,2^. Consequently, the threat of human impact on marine ecosystems and species is at an all-time high and continues to increase^1,3,4^. Impacts on marine ecosystems occur when they are under the influence of one or more stressors, often resulting from human activities. For example, overfishing is a stressor that may lead to fish population declines (see glossary of terms in Table 1).

**Table 1:** Glossary of terms and definitions.

Term	Definition
Stressor	A natural or anthropogenic pressure which causes a positive or negative quantifiable change in a response of the ecosystem^13^
Impact	The measurable effect of human activity on ecological condition^15^
Cumulative impacts	An estimate of the impact of multiple stressors^15^
Additive	An additive interaction type where the sum of the impact of individual stressors is used to calculate the cumulative impact^15^
Synergy	A non-additive interaction between stressors, where the cumulative impact is greater than the sum of the impact of individual stressors^14^. Above 0 indicates a positive synergy, below 0 indicates a negative synergy
Antagonistic	A non-additive interaction between stressors, where the cumulative impact is less than the sum of the impact of individual stressors^14^
Article	A published paper found through our literature searches
Study	A stressor combination on a seagrass response. A single article could have multiple studies if they tested multiple stressor combinations

There are many published studies that aim to understand the impact of stressors on marine ecosystems^5–8^. Recent meta-analyses have assessed interaction types between stressors in marine ecosystems, however many have only looked at specific stressor pairs, see Harvey et al.^9^, Jackson^10^ and Przeslavski et al.^11^, or have focussed on limited biological responses (e.g. Strain et al.^12^). Broader meta-analyses looking at many stressor combinations were undertaken in the past (for example, Crain et al.^13^^)^, but need to be updated due to the large number of studies that have been published since that time.

Accurately predicting and quantifying the impacts of stressors on marine environments is an important factor in establishing appropriate management and conservation strategies^14,15^. Inaccurate predictions of impact can potentially yield ‘ecological surprises’, which are unexpected changes in the natural environment^16^. Stressors rarely (if ever) occur in isolation, and the collective impact of multiple stressors is known as the cumulative impact (Table 1). It is important to know how stressors interact and how interactions affect the cumulative impact in order to inform management of marine ecosystems^17–19^.

Predicting the cumulative impact of multiple stressors from single stressor studies is only possible if stressors act independently of one another. This allows us to use an additive model to calculate the cumulative impact^20^, where we use the sum of the impact of individual stressors to indicate the combined overall impact. For example, if we have a quantified measure of change in biodiversity due to fishing, and the same measure of change, albeit at a different magnitude, due to pollution, we can sum these two measures of impact to estimate the cumulative impact of both stressors. However, this estimate will only be realistic if the stressors truly have independent effects on the biodiversity in the area. This assumption of additivity may not be appropriate. For example, synergies are common in marine ecosystems^21–23^ and occur when the total impact from multiple stressors is greater than what we might expect from an additive model^14^. This leads to an underestimate of the cumulative impact if using an additive model^11^, thereby increasing the chance of ecological surprises. Conversely, an antagonistic interaction occurs when the total impact of multiple stressors is less than what we expect based on an additive model^14^. For example, turbidity caused by run-off (stressor 1) could mitigate the effect of ultraviolet radiation (stressor 2) on benthic seagrass habitat by shading it^18^. Thus, the cumulative impact of turbidity and ultraviolet radiation may be less than what we would expect based on an assumption of additivity. Identifying interactions between multiple stressors is important to marine conservation and management as it presents an opportunity to achieve a larger benefit to an ecosystem by removing synergisms, whereas removing antagonisms may not be effective and could potentially worsen conditions. Conversely, where additive interactions are identified, stressors can be addressed individually without complex interactions needing to be considered^24^.

Seagrass ecosystems are some of the most productive on earth and provide many valuable ecosystem services^25–27^. These services include carbon sequestration^28^, supporting commercial fisheries^29^, water filtration, and protection from coastal erosion^27,30^. It is estimated that the resources and services provided by seagrass ecosystems contribute over US$100 million per year to the world’s blue economy^31^.

Unfortunately, seagrass ecosystems and the services they provide are under increasing stress from human impacts^26,32–34^. The effects of coastal development, climate change, and ecological degradation threaten seagrass ecosystems the world over^35,36^. Habitat fragmentation occurs when seagrass cover is reduced due to the adverse effect of coastal development or because of boating activity, for example^32,37^. Ocean warming and acidification are two well-known impacts that result from climate change^38,39^, which can increase herbivory pressure on seagrass^40^ and negatively affect carbon reserves^41^. Other stressors caused by human activity include increased nutrients and pollutants from run-off^42^ and invasive species presenting new challenges, such as increased competition or herbivory pressure^43^. To prevent further seagrass decline, we need to identify where and how often these stressors occur, if they interact, and the direction and magnitude of the impact^44^.

Seagrass ecosystems provide a case study to test the assumption of additivity in marine spatial cumulative impact assessment methods. The status of seagrasses as biological sentinels and the close association of seagrass to densely populated coastlines make them good indicators of anthropogenic stressors^45^. Further, the ecosystem services seagrasses provide give them high value and importance in management, and conservation and restoration strategies.

This study aims to establish how different stressor combinations interact in seagrass ecosystems. We conducted a meta-analysis using data from published studies of two or more stressors on seagrasses and classified each combination as either additive, synergistic, or antagonistic. We attempt to identify generalisations of stressor interactions on seagrasses and to test the assumption of additive effects of multiple stressors in cumulative impact assessment methods.

## Results

**Figure 1:**
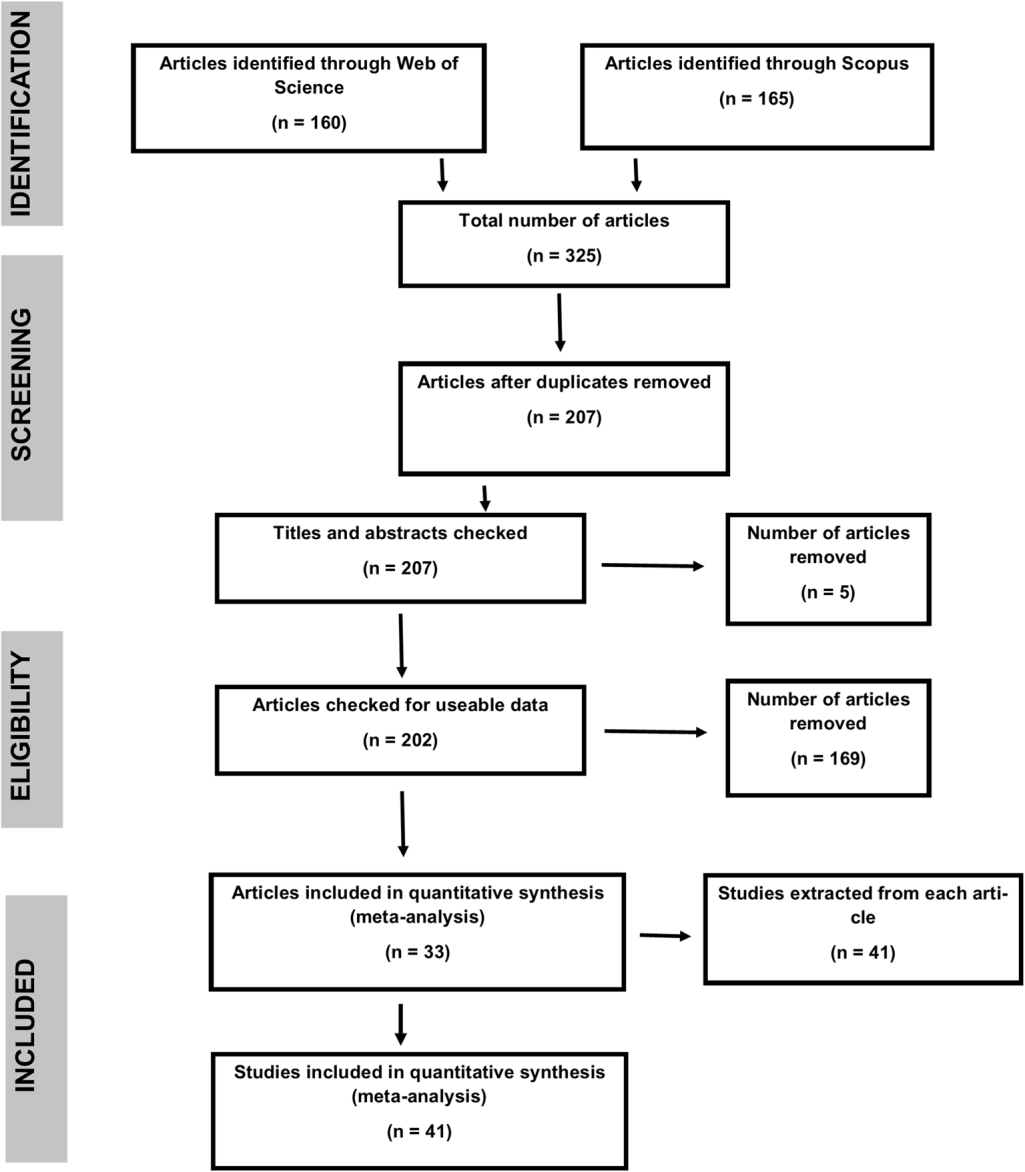
Article inclusions following a modified version of the PRISMA (Preferred Reporting Items for Systematic Review and Meta-Analysis) methodology^47^.

Across both searches, WoS returned 160 articles, and Scopus returned 165, for a total of 325 articles identified (Fig. 1). After duplicates were removed, we were left with 207 articles. Articles were removed if they were not relevant to this study, such as those looking at terrestrial or non-seagrass ecosystems (n = 116). Articles were also removed if the data required for calculating an effect size were not provided (n = 32). Some studies identified did not consider two or more stressors and so were omitted from our study (n = 26). In total, 201 stressor combinations from 41 studies taken from 33 articles were used for our meta-analysis.

**Figure 2:**
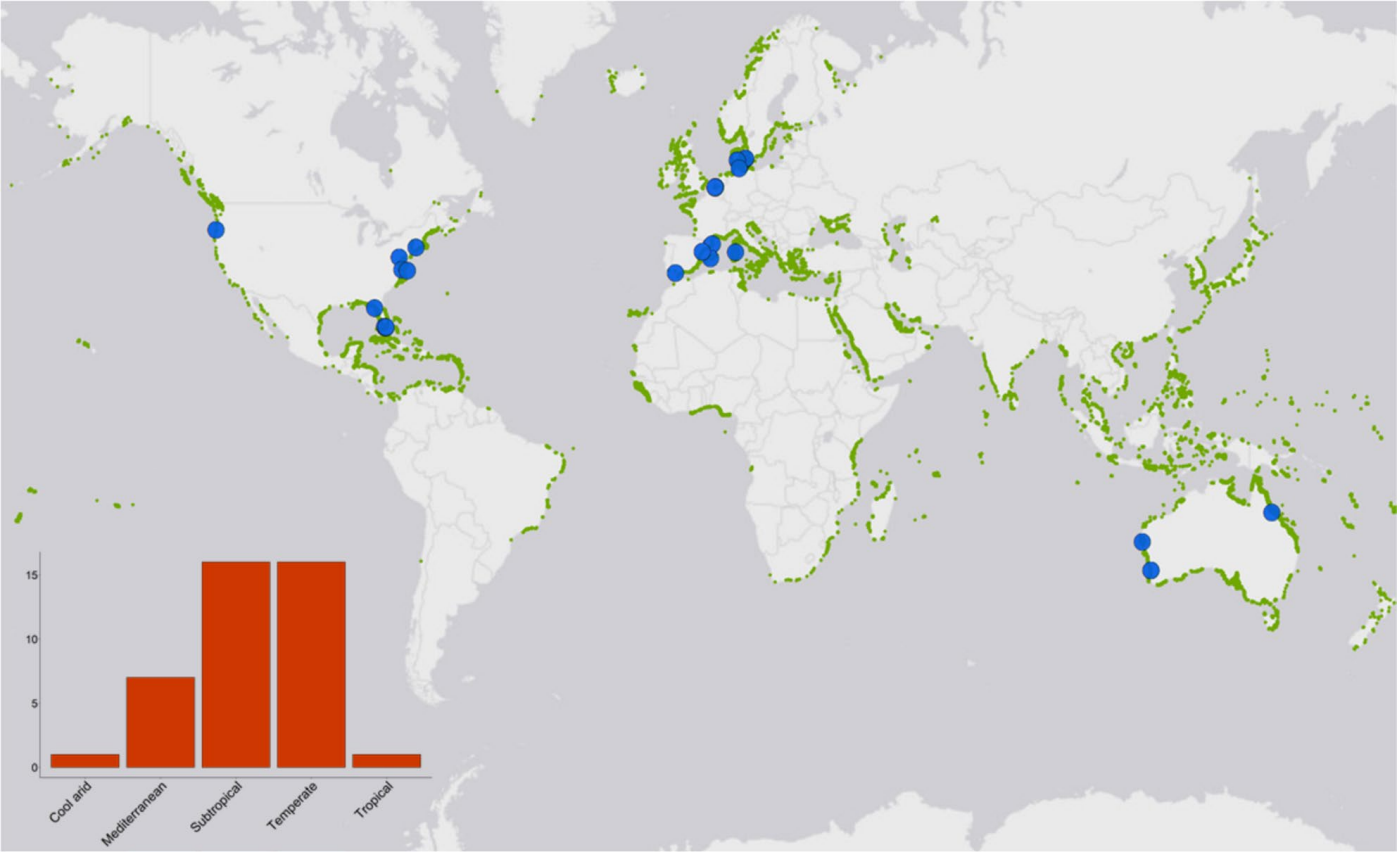
Map showing the location of studies used in our meta-analysis and the global distribution of seagrass^80^. Inset plot shows the count of studies depending on the broad climatic region where they were undertaken. Seagrass distribution data use layers ‘WCMC_013_014_SeagrassesPt_v6’ and ‘WCMC_013_014_SeagrassesPy_v6’, which can be accessed at https://gis.unep-wcmc.org/arcgis/rest/services/marine/WCMC_013_014_Seagrass_WMS/MapServer.

**Figure 3:**
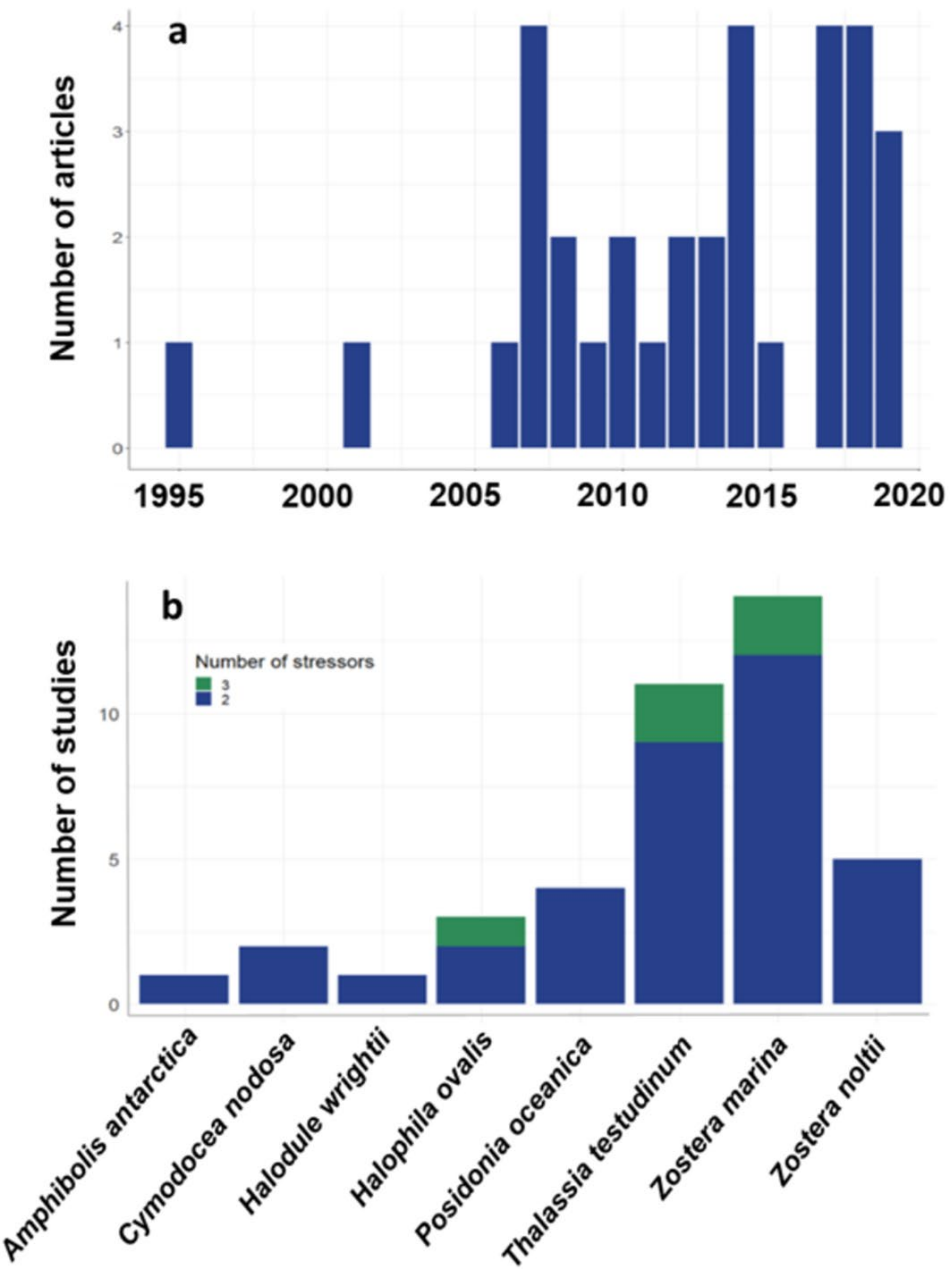
(**a**) Number of published articles over time, based on a search of published studies in Web of Science and Scopus. Search terms were all derivatives of the words ‘synergy’, ‘antagonistic’ and ‘additive’ and ‘seagrass’. (**b**) The frequency of multi-stressor experiments on each species of seagrass across all studies included in our meta-analysis.

The majority of studies were undertaken in a laboratory rather than a field setting (field = 7, lab = 34). Studies on temperate seagrass ecosystems were most common, followed by subtropical ecosystems (Fig. 2). Most studies used in this meta-analysis were undertaken in North-eastern America, Scandinavia and western Europe (Fig. 2). Published articles increased from 2006, however there have been peaks and troughs in publications through to the present day (Fig. 3a). *Zostera marina* and *Thalassia testudinum* were the most commonly studied species (Fig. 3b).

**Figure 4:**
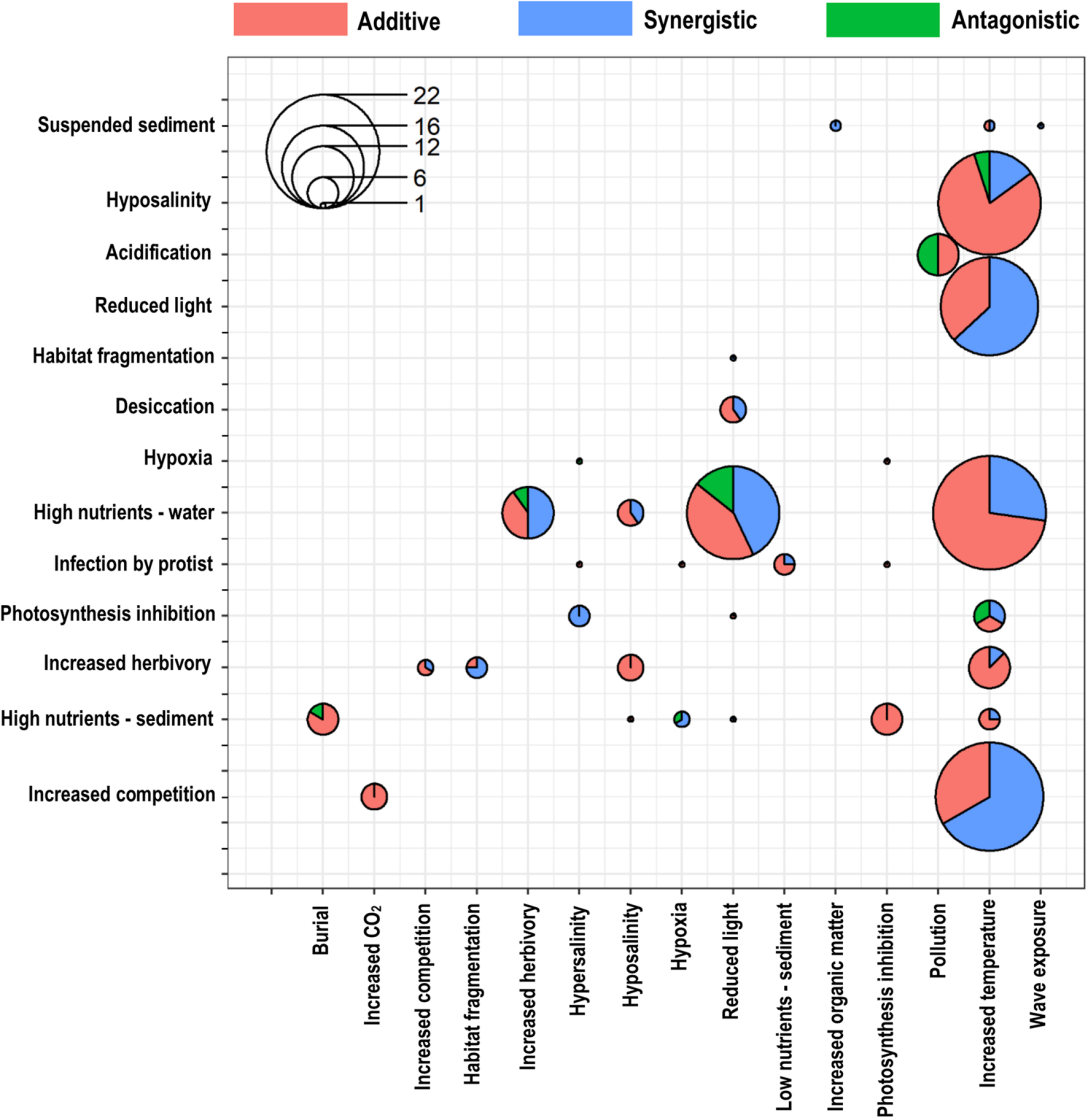
Plot to show the interaction type and number of tested stressor pairs. Points are sized according to the number of tested stressor pairs (i.e. larger points represent a higher number of tested pairs) and partitioned depending on the interaction type identified in each pair.

Studies rarely tested more than two stressors and we did not find any that tested more than three (Fig. 3b). We found useable data for 35 unique stressor pairs in total (for a full breakdown, see Supplementary Table S2 online). Increased temperature and increased nutrient levels were the most commonly tested stressor pair (22), followed closely by increased temperature and increased competition (21), and increased temperature and hyposalinity (20; Fig. 4). Other commonly-tested stressor pairs were reduced light and increased temperature (19) and reduced light and increased nutrients (18; Fig. 4; Supplementary Table S2 online).

Of the 201 tested stressor combinations, 115 of the interactions were additive, and 86 were non-additive including 73 synergisms, and 13 antagonisms (see Supplementary Table S3 online). Positive synergies were identified in 47 studies, and negative synergies in 26 studies. Due to the high number of studies testing two stressors, our analysis was mostly focussed around these. However, when high nutrient levels were introduced to a test of increased temperature and reduced light, the interaction switched from additive to a negative synergy (Supplementary Table S3 online).

## Discussion

In this study, we aimed to assess the validity of the assumption of additive stressor interactions which is used in many marine spatial cumulative impact assessment methods. Though additive interactions were most common in the seagrass studies we reviewed, synergies and antagonistic interactions were also frequently identified. This suggests that a blanket assumption of additivity in cumulative impact assessments is likely to over- or underestimate the impact of multiple stressors on seagrass ecosystems. Considering additivity is a general assumption in the cumulative impact assessment of many ecosystem types, more work evaluating the outputs of models which assume additivity is needed. Our results highlight the need for a better understanding of stressor interactions on various marine ecosystems to inform more realistic cumulative impact assessments verified with experimental data (for example Clark et al*.*^46^).

The most common interaction type we detected was an additive interaction (115 stressor interactions); however, there were some notable exceptions. When increased herbivory and habitat fragmentation were combined, many interactions were positively synergistic on the growth and biomass of seagrass (Supplementary Table S3 online). This suggests that when these stressors co-occur, growth and biomass of seagrass increases, which seems counter-intuitive since we expect both these stressors to have a detrimental impact on seagrass growth and biomass. However, it should be noted that this is based on a small number of studies (n = 3, with the fourth study reporting an additive interaction). There was a slightly higher number of both positive and negative synergistic interactions reported when increased herbivory was combined with high water nutrient levels, although additive interactions were also common. Exacerbation of stress by increased herbivory and high water nutrients can occur if the high nutrient content increases grazer population size^47^, or improves the nutritional quality of seagrass to grazers^48,49^. Failure to account for these synergies in cumulative impact assessments will result in an underestimate of impact, potentially causing misidentification of ecosystem thresholds. Côté et al*.*^17^ found that additive interactions were most common when increased herbivory and high water nutrients co-occurred in both terrestrial and marine ecosystems^18^. However, different studies that considered only the marine environment, report a mixture of antagonistic and synergistic interactions between increased herbivory and high water nutrients^50,51^. High nutrients is a particularly difficult stressor to predict as an increase in nutrients can have a positive impact up until a certain threshold, after which it can become toxic. This is an example of a non-linear response from an ecosystem to a stressor.

We found that positive synergies were most common when hypersalinity was combined with phytol stimulation in seagrass. Phytol is a compound that inhibits photosynthesis by degrading chloroplasts^52^. This contrasts to findings by Crain et al*.*^13^, who reported a higher number of antagonistic interactions between these stressors. Our results show that antagonistic interactions were most common when hypersalinity was paired with hypoxic conditions, which may well be one stressor mitigating another, or could be because the negative effect of one is so large that the second stressor seemingly has no effect^13,19^. Identifying where antagonistic interactions occur is important as the removal of one stressor may make little difference to ecosystem health or may even increase the impact of the other stressor. When photosynthesis-inhibiting toxins were added to hypersalinity and hypoxia, the interaction switched from antagonistic to a positive synergy on seagrass growth. This outcome was only detected in one study^53^ and may not be a general response, as hypersalinity has been reported to inhibit photosynthesis in some seagrass species such as *Thalassia testudinum*^54^. Therefore, there is little reason to expect that adding further photosynthesis-inhibiting toxins would increase the growth of seagrass^55–57^.

Reduced light and high water nutrient levels yielded a mixture of interaction types, which is consistent with results from Crain et al*.*^13^. However, it should be noted that Crain et al*.*^13^ looked at a range of marine ecosystems, not just seagrasses. We found that synergies were the most common interaction when increased temperature was combined with either reduced light or increased competition, although additive effects also occurred (in 28% of studies). These results are supported by previous meta-analyses, which also report a mixture of interactions between increased temperature and reduced light^13^, with antagonistic interactions occurring frequently^17^. Interactions between increased temperature and increased competition have been reported as additive in previous literature^58^. Increased temperature and hyposalinity was one of the most well-studied stressor combinations in our dataset, and we found a mixture of all three interaction types, with additive being the most common. This is consistent with other meta-analyses which also found that these stressors interacted differently depending on response or location^13,17,58^. Increased temperature and hyposalinity seems to be a difficult stressor combination to predict the effects of, with contrasting reports from various reviews and meta-analyses on different marine ecosystems. For example, Côté et al*.*^17^ found additive interactions between these stressors to be most common, whereas Darling and Côté^18^ found no additive interactions between these two stressors. However, it should be noted that Côté et al*.*^17^ focused on a broader range of ecosystems, including terrestrial, whereas Darling and Côté^18^ focused only on marine ecosystems. New research published in the 8 years between these studies may have also contributed to the differing results.

The most consistent result between our study and previous meta-analyses was the variation in interaction types detected across studies^11,13,17,18^, though none of these meta-analyses were specific to seagrass. Variation can be caused by a plethora of factors, which makes predicting all interactions extremely challenging without a large number of studies at regional scales^18^. Interactions between stressors can differ depending on the life history stage of the response organism^11^, though this was not explored in our study. Target species/ecosystems can also be a factor in differing interaction types^9^, for example, stressors that influence a species’ range may depend on the species being studied. Only one study in our meta-analysis compared the effect of a stressor combination on two different species of seagrass^59^. Koch et al*.*^59^ reported a mixture of interaction types between increased temperature and photosynthesis inhibition. Other studies that have tested stressor combinations across different species of macroalgae have reported a mixture of interaction types^60,61^. Though these studies were not on seagrass, the results suggest that further research across different species are needed if we are to make generalisations of stressor interaction types on all seagrass ecosystems. Depending on the seagrass genus being studied, we would expect different levels of resilience to stress and rates of recovery following disturbance. Resilience and recovery of seagrass would depend on the biology of that specific genus, including whether they are enduring and slow growing (*Posidonia*), or a transitory and fast-growing genus (*Halophila*)^62^.

Since we distinguish additive from non-additive interaction classification based on whether confidence intervals include 0, we looked at if non-additive interactions were more frequently detected in studies with a larger sample size. We looked at studies which used a sample size of greater than 10 and did not find a larger proportion of non-additive interactions. However, it should be noted that the majority of studies (81%) had a sample size of < 5.

The mixture of interaction types detected for the same stressor combinations across different studies suggests a need for location-specific cumulative impact assessment. Previous research on a freshwater ecosystem has highlighted the benefit of cumulative impact assessment methods which consider local stressor effects on specific ecosystem components present at the study location^63^. These results, and our work here, supports the idea that we cannot generalise how stressors interact across different ecosystems and regions.

Stressor combinations can interact differently depending on the latitude and climate of the study location^50^. The southern hemisphere is not well-represented here, with only 4 studies in our dataset (Fig. 2). Research by Burkepile and Hay^50^ found that the interactive effects of nutrient enrichment and increased herbivory on algae were opposite depending on if the algae was a temperate or tropical species. As with studies across different species, generalisations of stressor interactions across regions becomes more challenging when such variable results are found.

Variation in interaction types reflects the complexity and unpredictability of marine systems^64,65^. Complexity can be caused by a plethora of factors including ontogeny, spatial or temporal factors, as well as the pathways within trophic systems. These factors, and the complexity they cause, make it difficult to make generalities of stressor interactions across regions, organisms, species or life histories. Stressor interactions in many cumulative impact assessment methods are assumed to be additive^20^, and though the potential weakness of this assumption has been acknowledged for some time^64^, an additive model is still the most common when calculating cumulative impacts^66^.

Korpinen and Andersen^67^ reviewed 40 cumulative impact studies and found that 35 (88%) of the assessments assumed additive interactions. At present, the additive model is still the default for cumulative impact assessment methods despite the mounting evidence against it, though it should be noted that there are some published studies which do not assume additivity (for example Coll et al*.*^68^ and Griffith et al*.*^69^). This highlights the need for an evidence base on the appropriate use of stressor interaction types. Our data add to this evidence base, which can support a more nuanced approach to modelling marine spatial cumulative impacts that goes beyond the assumption of additivity and in doing so generates more realistic predictions of cumulative impacts for use in marine management^70^. Results presented here, and from previous reviews and meta-analyses^2,13^, suggest that cumulative impact methods based on the additive model should be interpreted with caution and their caveats clearly outlined. Whilst additive interactions are the most prevalent, non-additive interaction types are also common, suggesting that these should potentially be considered when calculating cumulative impacts. Prioritising experimental studies that test the combined effect of multiple stressors on different ecosystems and species (such as Clark et al*.*^46^ and Andersen et al*.*^71^) would help to fill gaps in the knowledge presented here.

Without more accurate predictions of stressor interactions, calculating reliable cumulative impact scores is challenging using existing modelling methods^72,73^. Marine ecosystems are complex environments with myriad factors seemingly altering stressor interactions from one ecosystem to another^9,18^ Future research could use the results from meta-analyses such as this one to re-calculate cumulative impact scores based on different and appropriate types of interactions between specific stressors, giving us a measure of impact, which can then be related to empirical condition data. Doing so could help us to understand how stressors are impacting marine ecosystems, and where removing stressors will provide the greatest benefit and help inform management of human-induced stressors and estimates of cumulative impacts.

## Methods

### Data collection

We conducted a literature search using the Web of Science (WoS) and Scopus search tools. Our search focused on seagrass ecosystems around the world. The initial literature search was undertaken on 10 October 2018. We searched the titles, abstracts and keywords of articles using the search terms: (synerg* OR antag* OR *additiv*) AND ‘seagrass’, where the asterisks represent all derivatives of the words ‘synergy’, ‘antagonistic’, and ‘additive’. These terms allowed us to cover all types of interactions (synergistic; antagonistic; non-additive/additive). We followed the preferred reporting items for systematic review and meta-analysis (PRISMA) protocol^74^ (Fig. 1).

A second search was undertaken on 11 June 2019 with the same search terms to update the results. Duplicates were removed following both searches. Titles and abstracts were checked for relevance to our study, and the articles then checked for useable data. Useable data here refers to the mean and variance of a control and treatment, including each stressor in isolation and the same stressors in combination.

To be included, each study needed to investigate the individual effect of two or more stressors, as well as their interactive effect (i.e. stressor 1; stressor 2; stressor 1 × 2). Articles which tested three or more stressors were treated as multiple, separate studies:

1. Stressor A vs. stressor B;
2. Stressor A vs. stressor C;
3. Stressor B vs. stressor C;
4. Stressor A vs. stressor B vs. stressor C.

Articles were subdivided into separate studies if the researchers tested different stressor combinations in the same paper (Table 1; Fig. 1). For example, if an article tested the effects of increased temperature and salinity on seagrass growth, and then increased temperature and nutrients on seagrass biomass, the article would be subdivided and treated as two studies as there were two response variables. Articles were also subdivided into separate studies if the researchers tested more than one level of the same stressor. For example, Kahn and Durako^75^ tested high and low nutrients against high and low salinity. Therefore, this article was treated as four separate studies:

1. High nutrients vs. high salinity;
2. Low nutrients vs. high salinity;
3. High nutrients vs. low salinity;
4. Low nutrients vs. low salinity.

If stressors were tested at multiple levels we only used the highest and lowest values^76^. Seagrass responses to each stressor pair were grouped into categories of impact for clearer interpretation and analysis. These categories were ‘Biodiversity’, ‘Biomass’, ‘Chemistry’, “Epiphytes’, ‘Growth’, ‘Mortality’, and ‘Survival’. ‘Biodiversity’ here refers to the number of organisms associated with seagrass.

### Effect size calculation

We used the standardised mean difference (SMD), also known as Hedge’s *d*^13^, and 95% confidence intervals to determine the effect size. We calculated the effect size for stressors acting in isolation, as well as the interactive effect between stressor combinations. For full details on how SMD was calculated see Supplementary Equation S1 online.

SMD uses the pooled sampling variance and a correction term to calculate and standardise the difference between the control and experimental means^77,78^. SMD has frequently been used as an effect size for factorial meta-analyses in ecology^13,76,78^, and uses an additive model, where deviation from this model signifies a non-additive interaction. The additive model is best suited for interpreting data from manipulative experiments^18,19^.

### Interaction classification

Three interaction types were classified here based on comparing the effect sizes of single and multi-stressor experiments (Table 1). Previous published studies have defined more interactions^18^, however we decided to use the main three as these are the most commonly and consistently defined. We followed the same definitions set out in Crain et al*.*^13^ and used by Lange et al*.*^76^, and we stated the direction (positive or negative) of the interaction when a synergy occurred.

**Figure 5:**
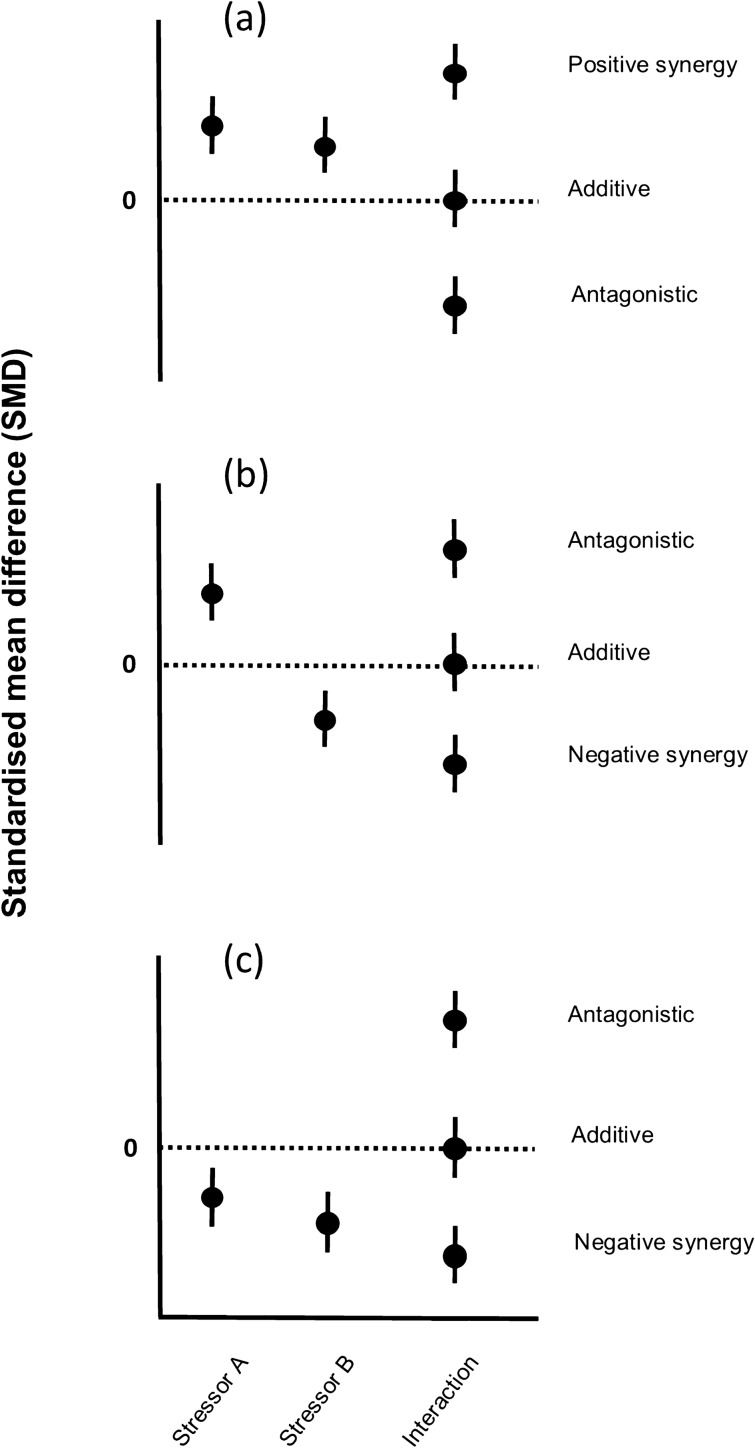
Conceptual schematic of interaction types, where the effect size of individual stressors (**A**,**B**) and the interaction are shown. Deviation from the additive model (Y = 0) represents a significant interaction. (**A**) Two positive individual effect sizes; (**B**) One positive, one negative individual effect sizes; (**C**) Two negative effect sizes^45^ (adapted from Crain et al. ^13^).

We used the interaction effect size (based on SMD) and the 95% confidence interval of this to determine interaction type. The interaction was considered additive if the 95% confidence intervals of the effect size included 0, signifying that the interaction is not significantly different from the sum of individual stressors^13,79^ (Fig. 5). When the individual effect sizes for all stressors were positive, an interaction effect size less than zero was classified as antagonistic, and an interaction effect size greater than zero was classified as synergistic (Fig. 5). When the individual effect sizes for all stressors were negative, interaction type was interpreted in the opposite manner (> 0 was antagonistic and < 0 was synergistic. For a visualisation of all effect size definitions, see Fig. 5).

## Supplementary information

Supplementary Information.
