# Supplementary material for: A meta-analysis of multiple stressors on seagrasses in the context of marine spatial cumulative impacts assessment
Source: Sci Rep. 2020 Jul 20;10:11934. doi: 10.1038/s41598-020-68801-w (PMC7371696; doi:10.1038/s41598-020-68801-w)
Supplement: Supplementary file 1 — Supplementary Information. [file 41598_2020_68801_MOESM1_ESM.pdf]

Supplementary material

TITLE: A meta-analysis of multiple stressors on seagrasses in the context of marine spatial cumulative impacts assessment

AUTHORS: Jackson Stockbridge[1], Alice R. Jones[1] & Bronwyn M. Gillanders[1]

ORCHiD ID:

Jackson Stockbridge: https://orcid.org/0000-0001-9905-8234

Alice R. Jones: https://orcid.org/0000-0002-6157-2024

Bronwyn M. Gillanders: https://orcid.org/0000-0002-7680-2240

AUTHOR ADDRESSES:

[1]Southern Seas Ecology Laboratories and Environment Institute, Darling Building DX 650 418, School of Biological Sciences, University of Adelaide, SA 5005 AUSTRALIA

EMAIL:

Jackson Stockbridge: Jackson.stockbridge@adelaide.edu.au

Alice R. Jones: alice.jones01@adelaide.edu.au

Bronwyn M. Gillanders: bronwyn.gillanders@adelaide.edu.au

Correspondence to: Jackson.stockbridge@adelaide.edu.au

Supplementary Equation S1. Calculations of standardised mean difference (SMD) as an effect size

SMD, or Hedge's *d*, was calculated as:

For stressor *A*:

$$d_A = \frac{(Y_A - Y_{ct})}{s} J(m)$$

For stressor *B*:

$$d_B = \frac{(Y_B - Y_{ct})}{s} J(m)$$

For interaction (*AB*):

$$d_{AB} = \frac{(Y_{AB} - Y_B) - (Y_A - Y_{ct})}{2s} J(m)$$

Where Y_{ct}, Y_A, Y_B and Y_{AB} are the mean responses of the control, stressor *A*, stressor *B* and the interactive effect of *A* and *B*, respectively. *S* is the pooled sampling variance and *J(m)* is a correction factor for small sample bias [1]. *J(m)* is calculated as:

$$J(m) = 1 - \frac{3}{4(N_{AB} + N_A + N_B + N_{ct} - 4) - 1}$$

Pooled sampling variance was calculated as:

$$S = \sqrt{\frac{(N_{AB} - 1)(\sigma_{AB}^2) + (N_A - 1)(\sigma_A^2) + (N_B - 1)(\sigma_B^2) + (N_{ct} - 1)(\sigma_{ct}^2)}{N_{AB} + N_A + N_B + N_{ct} - 4}}$$

Where *N* is the sample size and σ the standard deviation of each treatment.

Supplementary Table S2. Summary information of studies included in this meta-analysis including each stressor combination tested.

Study no.	Location	Climate	Species	Latitude	Longitude	Number of stressors	Response variable	Stressor 1	Stressor 2	Stressor 3	Reference
**1**	Portugal	Temperate	*Zostera noltii*	40.616	-8.738	2	Survival	Pollution	Acidification	-	2
**1**	Portugal	Temperate	*Zostera noltii*	40.616	-8.738	2	Biomass	Pollution	Acidification	-	2
**1**	Portugal	Temperate	*Zostera noltii*	40.616	-8.738	2	Growth	Pollution	Acidification	-	2
**2**	Mallorca	Mediterranean	*Posidonia oceanica*	39.35276	2.73665	2	Biomass	Temperature	Competition	-	3
**2**	Mallorca	Mediterranean	*Posidonia oceanica*	39.35276	2.73665	2	Mortality	Temperature	Competition	-	3
**2**	Mallorca	Mediterranean	*Posidonia oceanica*	39.35276	2.73665	2	Chemistry	Temperature	Competition	-	3
**2**	Mallorca	Mediterranean	*Posidonia oceanica*	39.35276	2.73665	2	Growth	Temperature	Competition	-	3
**3**	Alfacs Bay, NW Med	Mediterranean	*Cymodocea nodosa*	40.60495	0.68331	2	Chemistry	Temperature	Nutrient enrichment - water	-	4
**3**	Alfacs Bay, NW Med	Mediterranean	*Cymodocea nodosa*	40.60495	0.68331	2	Growth	Temperature	Nutrient enrichment - water	-	4
**3**	Alfacs Bay, NW Med	Mediterranean	*Cymodocea nodosa*	40.60495	0.68331	2	Mortality	Temperature	Nutrient enrichment - water	-	4
**4**	Alfacs Bay, NW Med	Mediterranean	*Cymodocea nodosa*	40.60495	0.68331	2	Chemistry	Temperature	Nutrient enrichment - sediment	-	4

Study no.	Location	Climate	Species	Latitude	Longitude	Number of stressors	Response variable	Stressor 1	Stressor 2	Stressor 3	Reference
**4**	Alfacs Bay, NW Med	Mediterranean	*Cymodocea nodosa*	40.60495	0.68331	2	Growth	Temperature	Nutrient enrichment - sediment	-	4
**4**	Alfacs Bay, NW Med	Mediterranean	*Cymodocea nodosa*	40.60495	0.68331	2	Mortality	Temperature	Nutrient enrichment - sediment	-	4
**5**	Denmark	Temperate	*Zostera marina*	55.7	11.78333	3	Growth	Temperature	Low light	Nutrient enrichment - water	5
**5**	Denmark	Temperate	*Zostera marina*	55.7	11.78333	3	Biomass	Temperature	Low light	Nutrient enrichment - water	5
**5**	Denmark	Temperate	*Zostera marina*	55.7	11.78333	3	Mortality	Temperature	Low light	Nutrient enrichment - water	5
**5**	Denmark	Temperate	*Zostera marina*	55.7	11.78333	3	Chemistry	Temperature	Low light	Nutrient enrichment - water	5
**6**	Sardinia	Mediterranean	*Posidonia oceanica*	40.56694	9.13472	2	Epiphytes	Burial	Nutrient enrichment - sediment	-	6
**6**	Sardinia	Mediterranean	*Posidonia oceanica*	40.56694	9.13472	2	Biomass	Burial	Nutrient enrichment - sediment	-	6
**6**	Sardinia	Mediterranean	*Posidonia oceanica*	40.56694	9.13472	2	Mortality	Burial	Nutrient enrichment - sediment	-	6
**6**	Sardinia	Mediterranean	*Posidonia oceanica*	40.56694	9.13472	2	Growth	Burial	Nutrient enrichment - sediment	-	6
**7**	Netherlands	Temperate	*Zostera noltii*	51.52778	3.94361	2	Biomass	Low light	Desiccation	-	7

Study no.	Location	Climate	Species	Latitude	Longitude	Number of stressors	Response variable	Stressor 1	Stressor 2	Stressor 3	Reference
**7**	Netherlands	Temperate	*Zostera noltii*	51.52778	3.94361	2	Growth	Low light	Desiccation	-	7
**8**	New York	Subtropical	*Zostera marina*	41.41667	-72.83333	2	Growth	CO2	Competition	-	8
**8**	New York	Subtropical	*Zostera marina*	41.41667	-72.83333	2	Chemistry	CO2	Competition	-	8
**9**	Queensland, Australia	Tropical	*Halophila ovalis*	-19.19111	146.85083	2	Growth	Temperature	Photosynthesis inhibition	-	9
**10**	Germany	Temperate	*Zostera marina*	54.65	10.3	2	Growth	Nutrient limitation - sediment	Infection	-	10
**10**	Germany	Temperate	*Zostera marina*	54.65	10.3	2	Biomass	Nutrient limitation - sediment	Infection	-	10
**11**	Florida	Subtropical	*Thalassia testudinum*	29.33333	-83.38333	2	Growth	Hypersalinity	Infection	-	11
**12**	Florida	Subtropical	*Thalassia testudinum*	29.33333	-83.38333	2	Growth	Temperature	Infection	-	11
**13**	Florida	Subtropical	*Thalassia testudinum*	29.33333	-83.38333	2	Growth	Photosynthesis inhibition	Infection	-	11
**14**	Florida	Subtropical	*Thalassia testudinum*	29.33333	-83.38333	2	Growth	Hypoxia	Infection	-	11
**15**	Denmark	Temperate	*Zostera marina*	55.48611	9.75556	2	Mortality	Light	Habitat modification	-	12
**16**	Denmark	Temperate	*Zostera marina*	55.71222	11.79306	2	Growth	Hyposalinity	Nutrient enrichment - water	-	13
**16**	Denmark	Temperate	*Zostera marina*	55.71222	11.79306	2	Biomass	Hyposalinity	Nutrient enrichment - water	-	13
**16**	Denmark	Temperate	*Zostera marina*	55.71222	11.79306	2	Mortality	Hyposalinity	Nutrient enrichment - water	-	13

Study no.	Location	Climate	Species	Latitude	Longitude	Number of stressors	Response variable	Stressor 1	Stressor 2	Stressor 3	Reference
**17**	Denmark	Temperate	*Zostera marina*	55.71222	11.79306	2	Mortality	Temperature	Hyposalinity	-	14
**17**	Denmark	Temperate	*Zostera marina*	55.71222	11.79306	2	Growth	Temperature	Hyposalinity	-	14
**17**	Denmark	Temperate	*Zostera marina*	55.71222	11.79306	2	Biomass	Temperature	Hyposalinity	-	14
**18**	Oregon, USA	Cool arid	*Zostera marina*	44.60639	-124.07472	2	Growth	Temperature	Nutrient enrichment - water	-	15
**19**	Netherlands	Temperate	*Zostera noltii*	51.65	4.01667	2	Biomass	Organic matter	Suspended sediment	-	16
**19**	Netherlands	Temperate	*Zostera noltii*	51.65	4.01667	2	Epiphytes	Organic matter	Suspended sediment	-	16
**20**	Shark Bay, WA	Temperate	*Amphibolis antarctica*	-25.93333	113.54222	2	Biomass	Temperature	Suspended sediment	-	17
**21**	Denmark	Temperate	*Zostera marina*	55.79456	11.77713	2	Growth	Low light	Nutrient enrichment - sediment	-	18
**21**	Denmark	Temperate	*Zostera marina*	55.79456	11.77713	2	Mortality	Low light	Nutrient enrichment - sediment	-	18
**21**	Denmark	Temperate	*Zostera marina*	55.79456	11.77713	2	Biomass	Low light	Nutrient enrichment - sediment	-	18
**21**	Denmark	Temperate	*Zostera marina*	55.79456	11.77713	2	Survival	Low light	Nutrient enrichment - sediment	-	18
**22**	Spain	Mediterranean	*Posidonia oceanica*	41.68417	2.81861	2	Biomass	Habitat modification	Herbivory	-	19

Study no.	Location	Climate	Species	Latitude	Longitude	Number of stressors	Response variable	Stressor 1	Stressor 2	Stressor 3	Reference
**22**	Spain	Mediterranean	*Posidonia oceanica*	41.68417	2.81861	2	Growth	Habitat modification	Herbivory	-	19
**22**	Spain	Mediterranean	*Posidonia oceanica*	41.68417	2.81861	2	Chemistry	Habitat modification	Herbivory	-	19
**23**	Netherlands	Temperate	*Zostera noltii*	51.56639	3.94906	2	Biomass	Wave exposure	Suspended sediment	-	20
**24**	Perth, WA	Temperate	*Halophila ovalis*	-32.00716	115.79298	3	Mortality	Temperature	Competition	Herbivory	21
**24**	Perth, WA	Temperate	*Halophila ovalis*	-32.00716	115.79298	3	Biomass	Temperature	Competition	Herbivory	21
**25**	Perth, WA	Temperate	*Halophila ovalis*	-31.98333	115.81667	2	Growth	Temperature	Competition	-	22
**25**	Perth, WA	Temperate	*Halophila ovalis*	-31.98333	115.81667	2	Biomass	Temperature	Competition	-	22
**26**	Virginia, USA	Subtropical	*Zostera marina*	39.52583	-77.05806	3	Epiphytes	Temperature	Hyposalinity	Herbivory	23
**26**	Virginia, USA	Subtropical	*Zostera marina*	39.52583	-77.05806	3	Biomass	Temperature	Hyposalinity	Herbivory	23
**26**	Virginia, USA	Subtropical	*Zostera marina*	39.52583	-77.05806	3	Growth	Temperature	Hyposalinity	Herbivory	23
**26**	Virginia, USA	Subtropical	*Zostera marina*	39.52583	-77.05806	3	Biodiversity	Temperature	Hyposalinity	Herbivory	23
**27**	Denmark	Temperate	*Zostera marina*	55.5	9.66667	2	Survival	Temperature	Competition	-	24
**28**	Denmark	Temperate	*Zostera marina*	55.51588	9.67549	2	Growth	Hypoxia	Nutrient enrichment - sediment	-	25
**28**	Denmark	Temperate	*Zostera marina*	55.51588	9.67549	2	Survival	Hypoxia	Nutrient enrichment - sediment	-	25

Study no.	Location	Climate	Species	Latitude	Longitude	Number of stressors	Response variable	Stressor 1	Stressor 2	Stressor 3	Reference
**29**	Germany	Temperate	*Zostera marina*	54.35	10.15	2	Epiphytes	Nutrient enrichment - water	Herbivory	-	26
**29**	Germany	Temperate	*Zostera marina*	54.35	10.15	2	Growth	Nutrient enrichment - water	Herbivory	-	26
**29**	Germany	Temperate	*Zostera marina*	54.35	10.15	2	Chemistry	Nutrient enrichment - water	Herbivory	-	26
**30**	Spain	Mediterranean	*Zostera noltii*	36.5	-6.16667	2	Growth	Low light	Nutrient enrichment - water	-	27
**31**	Spain	Mediterranean	*Posidonia oceanica*	42.04217	3.21434	2	Biomass	Nutrient enrichment - sediment	Photosynthesis inhibition	-	28
**31**	Spain	Mediterranean	*Posidonia oceanica*	42.04217	3.21434	2	Mortality	Nutrient enrichment - sediment	Photosynthesis inhibition	-	28
**31**	Spain	Mediterranean	*Posidonia oceanica*	42.04217	3.21434	2	Chemistry	Nutrient enrichment - sediment	Photosynthesis inhibition	-	28
**32**	Florida	Subtropical	*Thalassia testudinum*	25.04639	-80.75306	3	Growth	Photosynthesis inhibition	Hypersalinity	Hypoxia	29
**32**	Florida	Subtropical	*Thalassia testudinum*	25.04639	-80.75306	3	Survival	Photosynthesis inhibition	Hypersalinity	Hypoxia	29
**33**	Florida	Subtropical	*Thalassia testudinum*	24.92222	-80.7925	2	Growth	Temperature	Photosynthesis inhibition	-	30
**33**	Florida	Subtropical	*Thalassia testudinum*	24.92222	-80.7925	2	Biomass	Temperature	Photosynthesis inhibition	-	30
**34**	Florida	Subtropical	*Halodule wrightii*	25.22806	-80.79361	2	Growth	Temperature	Photosynthesis inhibition	-	30

Study no.	Location	Climate	Species	Latitude	Longitude	Number of stressors	Response variable	Stressor 1	Stressor 2	Stressor 3	Reference
**34**	Florida	Subtropical	*Halodule wrightii*	25.22806	-80.79361	2	Biomass	Temperature	Photosynthesis inhibition	-	30
**35**	Virginia, USA	Subtropical	*Zostera marina*	37.21667	-76.38333	2	Epiphytes	Nutrient enrichment - water	Herbivory	-	31
**35**	Virginia, USA	Subtropical	*Zostera marina*	37.21667	-76.38333	2	Biomass	Nutrient enrichment - water	Herbivory	-	31
**35**	Virginia, USA	Subtropical	*Zostera marina*	37.21667	-76.38333	2	Mortality	Nutrient enrichment - water	Herbivory	-	31
**35**	Virginia, USA	Subtropical	*Zostera marina*	37.21667	-76.38333	2	Biodiversity	Nutrient enrichment - water	Herbivory	-	31
**36**	Florida	Subtropical	*Thalassia testudinum*	25.0325	-80.50194	2	Growth	Nutrient limitation - water	Hyposalinity	-	32
**37**	Florida	Subtropical	*Thalassia testudinum*	25.0325	-80.50194	2	Growth	Nutrient enrichment - water	Hyposalinity	-	32
**38**	Florida	Subtropical	*Thalassia testudinum*	25.0325	-80.50194	2	Growth	Nutrient limitation - water	Hypersalinity	-	32
**39**	Florida	Subtropical	*Thalassia testudinum*	25.0325	-80.50194	2	Growth	Nutrient enrichment - water	Hypersalinity	-	32
**40**	Florida	Subtropical	*Thalassia testudinum*	25.125	-80.45083	3	Growth	Temperature	Hypersalinity	Photosynthesis inhibition	33
**40**	Florida	Subtropical	*Thalassia testudinum*	25.125	-80.45083	3	Biomass	Temperature	Hypersalinity	Photosynthesis inhibition	33
**41**	Virginia, USA	Subtropical	*Zostera marina*	37	-75	2	Growth	Low light	Photosynthesis inhibition	-	34

Supplementary Table S3. Summary of individual, combined and interaction type of each stressor and stressor combinations. When the individual effect sizes for all stressors were positive, an interaction effect size less than zero was classified as antagonistic, and if the interaction was more than 0 it was classified as synergistic (Fig. 5). For a visualisation of all effect size definitions, see Fig. 5.

Stressor A	Stressor B	Stressor C	Response variable	Individual effect A	Individual effect B	Individual effect C	Interaction effect	Interaction lower CI	Interaction upper CI	Interaction type	Direction (if synergistic)	Reference
Pollution	Acidification	-	Biomass	1.92	0	-	1.02	0.38	1.67	Antagonistic	-	2
Pollution	Acidification	-	Biomass	1.91	-0.443	-	1.95	1.21	2.68	Antagonistic	-	2
Light	High nutrients - water	-	Biomass	1.2	0.265	-	1.47	-0.332	3.28	Additive	-	5
Temperature	High nutrients - water	Light	Biomass	0.822	0.265	1.2	1.34	-0.43	3.11	Additive	-	5
Temperature	Light	-	Biomass	0.822	1.2	-	1.89	-0.0336	3.82	Additive	-	5
Temperature	High nutrients - water	-	Biomass	0.822	0.265	-	1.31	-0.452	3.08	Additive	-	5
Burial	High nutrients - sediment	-	Biomass	0.125	-0.417	-	0.616	-0.329	1.56	Additive	-	6
Burial	High nutrients - sediment	-	Biomass	0.451	-0.344	-	0.741	-0.214	1.7	Additive	-	6
Burial	High nutrients - sediment	-	Biomass	-0.461	-0.00376	-	-0.266	-1.19	0.662	Additive	-	6
Light	Desiccation	-	Biomass	0.587	1.08	-	1.08	-0.247	2.41	Additive	-	7
Light	Desiccation	-	Biomass	0.74	0.639	0.801		-0.488	2.09	Additive	-	7
Light	Desiccation	-	Biomass	0.62	1.31	-	1.42	0.0312	2.8	Synergistic	Positive	7
Light	Desiccation	-	Biomass	-0.437	0.644	-	-0.4	-1.65	0.852	Additive	-	7
Low nutrients - sediment	Infection	-	Biomass	1.64	1.39	-	2.58	0.413	4.74	Additive	-	10
Hyposalinity	High nutrients - water	-	Biomass	1.39	-1.67	-	-0.277	-1.89	1.33	Additive	-	13

Stressor A	Stressor B	Stressor C	Response variable	Individual effect A	Individual effect B	Individual effect C	Interaction effect	Interaction lower CI	Interaction upper CI	Interaction type	Direction (if synergistic)	Reference
Hyposalinity	Temperature	-	Biomass	0.3	0.0907	-	1.54	-0.285	3.36	Additive	-	14
Hyposalinity	Temperature	-	Biomass	-0.678	0.489	-	1.04	-0.669	2.74	Additive	-	14
Organic matter	Suspended sediment	-	Biomass	8.78	4.05	-	4.35	2.08	6.63	Synergistic	Positive	16
Temperature	Suspended sediment	-	Biomass	-0.893	1.03	-	0.147	-0.432	0.725	Additive	-	17
Temperature	Suspended sediment	-	Biomass	1.29	0.227	-	1.46	0.81	2.11	Synergistic	Positive	17
Light	High nutrients - water	-	Biomass	-0.571	-0.619	-	-2.64	-4.83	-0.453	Synergistic	Negative	18
Light	High nutrients - sediment	-	Biomass	0.296	0.28	-	4.79	1.64	7.94	Synergistic	Positive	18
Habitat modification	Herbivory	-	Biomass	0.8	1.16	-	2.46	1.3	3.62	Synergistic	Positive	19
Habitat modification	Herbivory	-	Biomass	0.746	-0.0897	-	-0.448	-1.34	0.439	Additive	-	19
Wave exposure	Suspended sediment	-	Biomass	0.95	1.08	-	1.25	0.371	2.12	Synergistic	Positive	20
Competition	Herbivory	-	Biomass	0.02	-0.0177	-	-0.602	-2.24	1.03	Additive	-	21
Temperature	Competition	Herbivory	Biomass	0	0.02	-0.0177	0.0436	-1.56	1.64	Additive	-	21
Temperature	Competition	-	Biomass	0	0.02	-	1.46	-0.342	3.26	Additive	-	21
Temperature	Herbivory	-	Biomass	0	-0.0177	-	0.748	-0.907	2.4	Additive	-	21
Temperature	Competition	-	Biomass	0.632	5.37	-	5.42	2.74	8.11	Synergistic	Positive	22
Temperature	Competition	-	Biomass	1.01	6.83	-	2.93	1.15	4.72	Synergistic	Positive	22
Temperature	Competition	-	Biomass	2.35	4.76	-	5.84	3	8.69	Synergistic	Positive	22
Hyposalinity	Herbivory	-	Biomass	0.697	0.606	-	0.483	-1.14	2.11	Additive	-	23
Temperature	Hyposalinity	Herbivory	Biomass	0.702	0.697	0.606	0.343	-1.27	1.95	Additive	-	23
Temperature	Hyposalinity	-	Biomass	0.702	0.697	-	0.435	-1.18	2.05	Additive	-	23

Stressor A	Stressor B	Stressor C	Response variable	Individual effect A	Individua l effect B	Individua l effect C	Interaction effect	Interaction lower CI	Interaction upper CI	Interaction type	Direction (if synergistic)	Reference
Temperature	Herbivory	-	Biomass	0.702	0.606	-	0.676	-0.969	2.32	Additive	-	23
Hyposalinity	Herbivory	-	Biomass	-0.242	-0.0175	-	0.171	-1.43	1.77	Additive	-	23
Temperature	Hyposalinity	Herbivory	Biomass	-0.0938	-0.242	-0.0175	0.0831	-1.52	1.68	Additive	-	23
Temperature	Hyposalinity	-	Biomass	-0.0938	-0.242	-	0.495	-1.13	2.12	Additive	-	23
Temperature	Herbivory	-	Biomass	-0.0938	-0.0175	-	0.572	-1.06	2.2	Additive	-	23
Photosynthesis inhibition	High nutrients – sediment	-	Biomass	2.05	NA	-	1.86	-0.0528	3.78	Additive	-	28
Photosynthesis inhibition	High nutrients - sediment	-	Biomass	0.601	NA	-	0.96	-0.73	2.65	Additive	-	28
Photosynthesis inhibition	High nutrients - sediment	-	Biomass	0.895	NA	-	-0.0412	-1.64	1.56	Additive	-	28
Herbivory	High nutrients - water	-	Biomass	4.24	2.15	-	1.76	0.304	3.23	Synergistic	Positive	31
Hyposalinity	Herbivory	-	Biodiversity	0.254	0.761	-	1.09	-0.625	2.81	Additive	-	23
Temperature	Hyposalinity	Herbivory	Biodiversity	0.334	0.254	0.761	0.775	-0.884	2.43	Additive	-	23
Temperature	Hyposalinity	-	Biodiversity	0.334	0.254	-	-0.249	-1.86	1.36	Additive	-	23
Temperature	Herbivory	-	Biodiversity	0.334	0.761	-	0.988	-0.707	2.68	Additive	-	23
Herbivory	High nutrients - water	-	Biodiversity	-0.292	-1.5	-	-0.0386	-1.28	1.2	Additive	-	31
Herbivory	High nutrients - water	-	Biodiversity	0.593	1.73	-	1.8	0.33	3.27	Synergistic	Positive	31
Herbivory	High nutrients - water	-	Biodiversity	0.456	1.1	-	0.957	-0.352	2.27	Additive	-	31
Herbivory	High nutrients - water	-	Biodiversity	-0.501	0.915	-	1.02	-0.297	2.34	Additive	-	31
Temperature	Competition	-	Chemistry	-1.21	-0.506	-	-0.704	-2.13	0.724	Additive	-	3
Temperature	Competition	-	Chemistry	-4.6	0.157	-	-2.38	-4.2	-0.571	Synergistic	Negative	3

Stressor A	Stressor B	Stressor C	Response variable	Individual effect A	Individual effect B	Individual effect C	Interaction effect	Interaction lower CI	Interaction upper CI	Interaction type	Direction (if synergistic)	Reference
Temperature	Competition	-	Chemistry	-0.862	0	-	-0.824	-2.27	0.62	Additive	-	3
Temperature	Competition	-	Chemistry	-1.33	-1.27	-	0.434	-0.968	1.84	Additive	-	3
Temperature	Competition	-	Chemistry	-3.62	-0.424	-	-3.17	-5.25	-1.09	Synergistic	Negative	3
Temperature	High nutrients - water	-	Chemistry	-3.54	-1.65	-	-1.18	-2.91	0.557	Additive	-	4
Temperature	High nutrients - water	-	Chemistry	-0.531	-1.98	-	-1.28	-3.04	0.474	Additive	-	4
Temperature	High nutrients – water	-	Chemistry	-0.958	-1.05	-	-1.72	-0.359	0.155	Additive	-	4
Temperature	High nutrients - sediment	-	Chemistry	-0.718	0.105	-	-1.79	-3.68	0.105	Additive	-	4
Temperature	High nutrients - sediment	-	Chemistry	0.868	0.964	-	-0.695	-2.34	0.953	Additive	-	4
Temperature	High nutrients - sediment	-	Chemistry	1	-0.767	-	-1.3	-3.06	0.463	Additive	-	4
Light	High nutrients - water	-	Chemistry	8.24	8.06	-	9.5	3.89	15.1	Synergistic	Positive	5
Temperature	Light	High nutrients - water	Chemistry	4.55	8.24	8.06	9.41	3.85	15	Synergistic	Positive	5
Temperature	Light	-	Chemistry	4.55	8.24	-	8.65	3.5	13.8	Synergistic	Positive	5
Temperature	High nutrients - water	-	Chemistry	4.55	8.06	-	8.66	3.5	13.8	Synergistic	Positive	5
Light	High nutrients - water	-	Chemistry	2.23	3.1	-	3.12	0.739	5.51	Synergistic	Positive	5
Temperature	Light	High nutrients - water	Chemistry	1.85	2.23	3.1	2.89	0.6	5.17	Synergistic	Positive	5
Temperature	Light	-	Chemistry	1.85	2.23	-	2.43	0.322	4.54	Synergistic	Positive	5
Temperature	High nutrients - water	-	Chemistry	1.85	3.1	-	3.38	0.884	5.87	Synergistic	Positive	5

Stressor A	Stressor B	Stressor C	Response variable	Individual effect A	Individual effect B	Individual effect C	Interaction effect	Interaction lower CI	Interaction upper CI	Interaction type	Direction (if synergistic)	Reference
Light	High nutrients - water	-	Chemistry	-1.43	-5.14	-	-8.61	-13.7	-3.48	Synergistic	Negative	5
Temperature	Light	High nutrients - water	Chemistry	-0.634	-1.43	-5.14	-3.98	-6.74	-1.22	Synergistic	Negative	5
Temperature	Light	-	Chemistry	-0.634	-1.43	-	-1.7	-3.57	0.167	Additive	-	5
Temperature	High nutrients - water	-	Chemistry	-0.634	-5.14	-	-3.52	-6.08	-0.966	Synergistic	Negative	5
Light	High nutrients – water	-	Chemistry	-2.29	-2.24	-	-2.79	-5.04	-0.544	Synergistic	Negative	5
Temperature	Light	High nutrients - water	Chemistry	-0.712	-2.29	-2.24	-1.23	-2.98	0.512	Additive	-	5
Temperature	Light	-	Chemistry	-0.712	-2.29	-	-1.45	-3.25	0.35	Additive	-	5
Temperature	High nutrients - water	-	Chemistry	-0.712	-2.24	-	-0.579	-2.21	1.05	Additive	-	5
CO2	Competition	-	Chemistry	-1.31	-0.412	-	-1.13	-2.85	0.593	Additive	-	8
CO2	Competition	-	Chemistry	0.461	0.921	-	0.921	-0.762	2.6	Additive	-	8
Habitat modification	Herbivory	-	Chemistry	1.74	0.194	-	1.79	0.756	2.83	Synergistic	Positive	19
Herbivory	High nutrients - water	-	Chemistry	-0.175	3.21	-	2.26	0.213	4.31	Antagonistic	-	26
Photosynthesis inhibition	High nutrients - sediment	-	Chemistry	0.0269	NA	-	-0.349	-1.96	1.26	Additive	-	28
Photosynthesis inhibition	High nutrients - sediment	-	Chemistry	0.287	NA	-	0.0048	-1.6	1.61	Additive	-	28
Photosynthesis inhibition	High nutrients - sediment	-	Chemistry	0.772	NA	-	1.17	-0.56	2.9	Additive	-	28
Burial	High nutrients - sediment	-	Epiphytes	0.426	0.148	-	-1.7	-2.78	-0.621	Antagonistic	-	6
Organic matter	Suspended sediment	-	Epiphytes	-4.6	-5.31	-	-5.16	-7.74	-2.58	Synergistic	Positive	16
Hyposalinity	Herbivory	-	Epiphytes	0.694	0.694	-	0.685	-0.962	2.33	Additive	-	23

Stressor A	Stressor B	Stressor C	Response variable	Individual effect A	Individual effect B	Individual effect C	Interaction effect	Interaction lower CI	Interaction upper CI	Interaction type	Direction (if synergistic)	Reference
Temperature	Hyposalinity	Herbivory	Epiphytes	0.404	0.626	0.694	0.614	-1.02	2.25	Additive	-	23
Temperature	Hyposalinity	-	Epiphytes	0.404	0.626	-	0.812	-0.853	2.48	Additive	-	23
Temperature	Herbivory	-	Epiphytes	0.404	0.694	-	0.682	-0.964	2.33	Additive	-	23
Hypoxia	High nutrients - sediment	-	Epiphytes	0	-1.05	-	-5.83	-9.49	-2.16	Synergistic	Negative	25
Herbivory	High nutrients - water	-	Epiphytes	4.25	-21.5	-	-14.3	-22.5	-6.05	Synergistic	Negative	26
Herbivory	High nutrients - water	-	Epiphytes	-1.47	0.639	-	-1.03	-2.35	0.291	Synergistic	Negative	31
Pollution	Acidification	-	Growth	0.895	0.356	-	0.447	-0.165	1.06	Additive	-	2
Pollution	Acidification	-	Growth	2.37	-0.103	-	1.44	0.76	2.12	Antagonistic	-	2
Pollution	Acidification	-	Growth	1.26	-0.0427	-	0.683	0.0606	1.31	Antagonistic	-	2
Pollution	Acidification	-	Growth	1.75	0.72	-	-1.17	-2.39	0.0556	Additive	-	2
Pollution	Acidification	-	Growth	2.9	0	-	0.362	-0.779	1.5	Additive	-	2
Temperature	Competition	-	Growth	-0.631	-0.721	-	-0.239	-1.63	1.15	Additive	-	3
Temperature	High nutrients - water	-	Growth	2.89	-0.509	-	3.02	0.676	5.36	Synergistic	Positive	4
Temperature	High nutrients - water	-	Growth	2.07	0.128	-	1.89	-0.0371	3.81	Additive	-	4
Temperature	High nutrients - sediment	-	Growth	0.439	-1.04	-	1.51	-0.305	3.32	Additive	-	4
Temperature	High nutrients - sediment	-	Growth	9.71	2.08	-	6.15	2.32	9.98	Synergistic	Positive	4
Temperature	High nutrients - sediment	-	Growth	-0.755	-0.195	-	1.38	-0.403	3.16	Additive	-	4
Light	High nutrients - water	-	Growth	1.66	1.01	-	3.09	0.719	5.46	Synergistic	Positive	5

Stressor A	Stressor B	Stressor C	Response variable	Individual effect A	Individual effect B	Individual effect C	Interaction effect	Interaction lower CI	Interaction upper CI	Interaction type	Direction (if synergistic)	Reference
Temperature	Light	High nutrients - water	Growth	2.63	1.66	1.01	7.01	2.73	11.3	Synergistic	Positive	5
Temperature	Light	-	Growth	2.63	1.66	-	5.55	2.03	9.07	Synergistic	Positive	5
Temperature	High nutrients - water	-	Growth	2.63	1.01	-	4.95	1.72	8.17	Synergistic	Positive	5
Light	High nutrients - water	-	Growth	2.32	1.44	-	1.25	-0.497	3	Additive	-	5
Temperature	Light	High nutrients - water	Growth	1.44	2.32	1.44	3.41	0.902	5.91	Synergistic	Positive	5
Temperature	Light	-	Growth	1.44	2.32	-	3.25	0.815	5.69	Synergistic	Positive	5
Temperature	High nutrients - water	-	Growth	1.44	1.44	-	0.649	-0.993	2.29	Additive	-	5
Light	High nutrients – water	-	Growth	2.81	1.18	-	2.02	0.0512	3.98	Synergistic	Positive	5
Temperature	Light	High nutrients - water	Growth	1.99	2.81	1.18	4.81	1.65	7.96	Synergistic	Positive	5
Temperature	Light	-	Growth	1.99	2.81	-	2.68	0.476	4.89	Synergistic	Positive	5
Temperature	High nutrients - water	-	Growth	1.99	1.18	-	1.39	-0.394	3.17	Additive	-	5
Light	High nutrients - water	-	Growth	1.2	-0.37	-	0.206	-1.4	1.81	Additive	-	5
Temperature	Light	High nutrients - water	Growth	-0.0375	1.2	-0.37	1.4	-0.387	3.18	Additive	-	5
Temperature	Light	-	Growth	-0.0375	1.2	-	0.881	-0.795	2.56	Additive	-	5
Temperature	High nutrients - water	-	Growth	-0.0375	-0.37	-	0.667	-0.977	2.31	Additive	-	5
Burial	High nutrients - sediment	-	Growth	0.116	-0.772	-	-0.964	-1.94	0.012	Additive	-	6

Stressor A	Stressor B	Stressor C	Response variable	Individual effect A	Individual effect B	Individual effect C	Interaction effect	Interaction lower CI	Interaction upper CI	Interaction type	Direction (if synergistic)	Reference
Light	Desiccation	-	Growth	0.606	1.01	-	1.53	0.122	2.94	Synergistic	Positive	7
CO2	Competition	-	Growth	-0.736	0.106	-	-0.417	-2.03	1.2	Additive	-	8
CO2	Competition	-	Growth	-0.854	-0.0678	-	-0.679	-2.33	0.966	Additive	-	8
CO2	Competition	-	Growth	-1.37	0.361	-	0.181	-1.42	1.78	Additive	-	8
Temperature	Photosynthesis inhibition	-	Growth	-1.74	-8.87	-	-8.24	11.1	-5.39	Synergistic	Negative	9
Low nutrients - sediment	Infection	-	Growth	1.5	0.0986	-	2.11	0.111	4.1	Synergistic	Positive	10
Low nutrients - sediment	Infection	-	Growth	0.767	1.52	-	1.65	-0.203	3.5	Additive	-	10
Low nutrients - sediment	Infection	-	Growth	-0.563	-0.439	-	-0.436	-2.06	1.18	Additive	-	10
Hypersalinity	Infection	-	Growth	-3.57	1.08	-	0.744	-0.538	2.03	Additive	-	11
Temperature	Infection	-	Growth	-0.642	0.713	-	0.707	-0.57	1.99	Additive	-	11
Photosynthesis inhibition	Infection	-	Growth	0.128	0.53	-	0.754	-0.529	2.04	Additive	-	11
Hypoxia	Infection	-	Growth	-0.336	0.675	-	0.692	-0.584	1.97	Additive	-	11
Hyposalinity	High nutrients - water	-	Growth	-0.793	-2.28	-	-0.985	-2.68	0.71	Additive	-	13
Hyposalinity	High nutrients - water	-	Growth	-0.369	0.109	-	0.00819	-1.59	1.61	Additive	-	13
Hyposalinity	High nutrients - water	-	Growth	1.89	-6.14	-	-2.21	-4.24	-0.179	Synergistic	Negative	13
Hyposalinity	High nutrients - water	-	Growth	1.37	-1.79	-	-2.09	-4.08	-0.0983	Synergistic	Negative	13
Hyposalinity	Temperature	-	Growth	2.25	-0.293	-	5.34	1.92	8.77	Antagonistic	-	14
Hyposalinity	Temperature	-	Growth	0.857	0.0932	-	5.93	2.21	9.65	Synergistic	Positive	14
Hyposalinity	Temperature	-	Growth	0.391	-0.23	-	1.73	-0.146	3.61	Additive	-	14
Hyposalinity	Temperature	-	Growth	-0.696	0.354	-	1.79	-0.106	3.68	Additive	-	14

Stressor A	Stressor B	Stressor C	Response variable	Individual effect A	Individual effect B	Individual effect C	Interaction effect	Interaction lower CI	Interaction upper CI	Interaction type	Direction (if synergistic)	Reference
Temperature	High nutrients - water	-	Growth	-0.844	-0.54	-	-0.925	-2.61	0.759	Additive	-	15
Temperature	High nutrients - water	-	Growth	-0.642	-0.216	-	-1.24	-2.98	0.509	Additive	-	15
Light	High nutrients - water	-	Growth	-1.38	-0.859	-	-0.639	-2.28	1	Additive	-	14
Light	High nutrients - water	-	Growth	-0.327	-0.378	-	1.81	-0.0885	3.71	Additive	-	14
Light	High nutrients - water	-	Growth	-1.65	-2.27	-	0.618	-1.02	2.26	Additive	-	14
Light	High nutrients - water	-	Growth	0.739	-0.077	-	4.92	1.71	8.13	Antagonistic	-	14
Habitat modification	Herbivory	-	Growth	1.71	1.25	-	2.33	1.19	3.46	Synergistic	Positive	19
Temperature	Competition	-	Growth	0.417	4.07	-	3.32	1.41	5.24	Synergistic	Positive	22
Temperature	Competition	-	Growth	0.874	4.78	-	5.29	2.66	7.92	Synergistic	Positive	22
Temperature	Competition	-	Growth	2.07	4.03	-	3.69	1.65	5.73	Synergistic	Positive	22
Temperature	Competition	-	Growth	1.63	2.23	-	1.12	1.72	5.87	Synergistic	Positive	22
Hyposalinity	Herbivory	-	Growth	0.679	0.628	-	0.348	-1.26	1.96	Additive	-	23
Temperature	Hyposalinity	Herbivory	Growth	0.792	0.679	0.628	-0.0127	-1.61	1.59	Additive	-	23
Temperature	Hyposalinity	-	Growth	0.792	0.679	-	0.474	-1.15	2.1	Additive	-	23
Temperature	Herbivory	-	Growth	0.792	0.628	-	0.371	-1.24	1.98	Additive	-	23
Hypoxia	High nutrients - sediment	-	Growth	0.738	2.91	-	6.99	2.72	11.3	Synergistic	Positive	25
Herbivory	High nutrients - water	-	Growth	-9.93	1.46	-	-2.93	-5.23	-0.626	Synergistic	Negative	26
Light	High nutrients - water	-	Growth	14.1	-6.32	-	4.24	1.36	7.13	Antagonistic	-	27
Hypersalinity	Photosynthesis inhibition	Hypoxia	Growth	1.82	1.16	-0.547	3.01	0.984	5.03	Synergistic	Positive	29

Stressor A	Stressor B	Stressor C	Response variable	Individual effect A	Individual effect B	Individual effect C	Interaction effect	Interaction lower CI	Interaction upper CI	Interaction type	Direction (if synergistic)	Reference
Hypersalinity	Photosynthesis inhibition	-	Growth	1.82	1.16	-	2.4	0.581	4.22	Synergistic	Positive	29
Hypersalinity	Hypoxia	-	Growth	1.82	-0.547	-	4.37	1.82	6.92	Antagonistic	-	29
Photosynthesis inhibition	Hypoxia	-	Growth	1.16	-0.547	-	-0.134	-1.52	1.25	Additive	-	29
Hypersalinity	Photosynthesis inhibition	-	Growth	5.83	2.56	-	6.25	2.89	9.62	Synergistic	Positive	29
Temperature	Photosynthesis inhibition	-	Growth	3.06	1.72	-	3.39	1.23	5.56	Synergistic	Positive	30
Temperature	Photosynthesis inhibition	-	Growth	-0.449	-0.96	-	1.47	0.0908	3.03	Additive	-	30
Temperature	Photosynthesis inhibition	-	Growth	3.13	-0.147	-	2.34	0.541	4.14	Antagonistic	-	30
Temperature	Photosynthesis inhibition	-	Growth	-0.385	0.767	-	8.62	4.18	13.1	Antagonistic	-	30
Temperature	Photosynthesis inhibition	-	Growth	0.445	1.92	-	0.631	-0.789	2.05	Additive	-	30
Light	Photosynthesis inhibition	-	Growth	0.147	0.0179	-	0.184	-1.43	1.78	Additive	-	34
Temperature	Competition	-	Mortality	-8.61	-0.699	-	-4.55	-7.18	-1.93	Synergistic	Negative	3
Temperature	High nutrients - water	-	Mortality	-0.661	-0.604	-	-1.71	-3.58	0.161	Additive	-	4
Temperature	High nutrients - sediment	-	Mortality	-5.02	-0.491	-	-2.33	-4.41	-0.259	Synergistic	Negative	4
Light	High nutrients - water	-	Mortality	-0.543		-	-1.25	-3	0.499	Additive	-	5
Temperature	Light	High nutrients - water	Mortality	-0.547	-0.543	-	-2.68	-4.88	-0.474	Synergistic	Negative	5
Temperature	Light	-	Mortality	-0.547	-0.543	-	-0.805	-2.47	0.859	Additive	-	5

Stressor A	Stressor B	Stressor C	Response variable	Individual effect A	Individual effect B	Individual effect C	Interaction effect	Interaction lower CI	Interaction upper CI	Interaction type	Direction (if synergistic)	Reference
Temperature	High nutrients - water	-	Mortality	-0.547		-	-0.965	-2.66	0.726	Additive	-	5
Burial	High nutrients - sediment	-	Mortality	-1.15	-0.849	-	-0.869	-1.84	0.0975	Additive	-	6
Light	Habitat modification	-	Mortality	-1.79	0.0202	-	-10.3	-14.6	-6.04	Synergistic	Negative	12
Hyposalinity	High nutrients - water	-	Mortality	-1.2	-0.98	-	-0.741	-2.39	0.914	Additive	-	13
Hyposalinity	Temperature	-	Mortality	-0.0739	-0.387	-	-2.21	-4.24	0.18	Synergistic	Negative	14
Hyposalinity	Temperature	-	Mortality	-0.000242	-0.0694	-	-0.44	-2.06	1.18	Additive	-	14
Hyposalinity	Temperature	-	Mortality	-0.0212	-0.675	-	-2.78	-5.2	-0.535	Synergistic	Negative	14
Hyposalinity	Temperature	-	Mortality	-0.358	-0.61	-	-1.08	-2.8	0.632	Additive	-	14
Light	High nutrients - water	-	Mortality	0.624	-2.3	-	-4.14	-6.97	-1.3	Synergistic	Negative	14
Competition	Herbivory	-	Mortality	-5.02	-0.332	-	-2.73	-4.95	-0.505	Synergistic	Negative	21
Temperature	Competition	Herbivory	Mortality	-1.42	-5.02	-0.332	-3.32	-5.79	-0.854	Synergistic	Negative	21
Temperature	Competition	-	Mortality	-1.42	-5.02	-	-2.95	-5.26	-0.637	Synergistic	Negative	21
Temperature	Herbivory	-	Mortality	-1.42	-0.332	-	-2.34	-4.41	-0.261	Synergistic	Negative	21
Competition	Herbivory	-	Mortality	-1.42	0.236	-	-1.52	-3.33	0.298	Additive	-	21
Temperature	Competition	Herbivory	Mortality	-0.733	-1.42	0.236	-1.67	-3.52	0.191	Additive	-	21
Temperature	Competition	-	Mortality	-0.733	-1.42	-	-1.96	-3.9	-0.00982	Synergistic	Negative	21
Temperature	Herbivory	-	Mortality	-0.733	0.236	-	-1.38	-3.16	0.403	Additive	-	21
Herbivory	High nutrients - water	-	Mortality	-1.54	-1.21	-	-1.22	-2.56	0.134	Additive	-	31
Pollution	Acidification	-	Survival	0	-0.291	-	0	-1.13	1.13	Additive	-	2
Light	High nutrients - water	-	Survival	-0.651	0.519	-	0.0698	-1.53	1.67	Additive	-	14
Temperature	Competition	-	Survival	1.86	0.595	-	5.47	1.99	8.95	Synergistic	Positive	24

Stressor A	Stressor B	Stressor C	Response variable	Individual effect A	Individual effect B	Individual effect C	Interaction effect	Interaction lower CI	Interaction upper CI	Interaction type	Direction (if synergistic)	Reference
Hypoxia	High nutrients - sediment	-	Survival	-0.048	1.63	-	2.64	0.449	4.82	Antagonistic	-	25
Hypersalinity	Photosynthesis inhibition	-	Survival	2.99	1.22	-	3.34	1.2	5.49	Synergistic	Positive	29

References

1 Hedges, L. V. & Olkin, I. *Statistical methods for meta-analysis*. (Academic press, 1985).

2 de los Santos, C., Arenas, F., Neuparth, T. & Santos, M. M. Interaction of short-term copper pollution and ocean acidification in seagrass ecosystems: Toxicity, bioconcentration and dietary transfer. *Mar. Pollut. Bull.* **142**, 155-163 (2019).

3 Pereda-Briones, L., Terrados, J. & Tomas, F. Negative effects of warming on seagrass seedlings are not exacerbated by invasive algae. *Mar. Pollut. Bull.* **141**, 36-45, doi:10.1016/j.marpolbul.2019.01.049 (2019).

4 Ontoria, Y. *et al.* Interactive effects of global warming and eutrophication on a fast-growing Mediterranean seagrass. *Marine Environmental Research* **145**, 27-38, doi:10.1016/j.marenvres.2019.02.002 (2019).

5 Moreno-Marin, F., Brun, F. G. & Pedersen, M. F. Additive response to multiple environmental stressors in the seagrass *Zostera marina* L. *Limnology and Oceanography* **63**, 1528-1544, doi:10.1002/lno.10789 (2018).

6 Ceccherelli, G. *et al.* Seagrass collapse due to synergistic stressors is not anticipated by phenological changes. *Oecologia* **186**, 1137-1152, doi:10.1007/s00442-018-4075-9 (2018).

7 Suykerbuyk, W. *et al.* Living in the intertidal: desiccation and shading reduce seagrass growth, but high salinity or population of origin have no additional effect. *Peerj* **6**, 5234, doi:10.7717/peerj.5234 (2018).

8 Young, C. S., Peterson, B. J. & Gobler, C. J. The bloom-forming macroalgae, *Ulva*, outcompetes the seagrass, *Zostera marina*, under high CO_2 conditions. *Estuar. Coasts* **41**, 1-16, doi:10.1007/s12237-018-0437-0 (2018).

9 Wilkinson, A. D. *et al.* Combined effects of temperature and the herbicide diuron on Photosystem II activity of the tropical seagrass *Halophila ovalis*. *Sci. Rep.* **7**, 45404, doi:10.1038/srep45404 (2017).

10 Brakel, J., Reusch, T. B. H. & Bockelmann, A. C. Moderate virulence caused by the protist *Labyrinthula zosterae* in ecosystem foundation species *Zostera marina* under nutrient limitation. *Mar. Ecol. Prog. Ser.* **571**, 97-108, doi:10.3354/meps12104 (2017).

11 Bishop, N., Martin, D. L. & Ross, C. Effects of multi-stress exposure on the infection dynamics of a *Labyrinthula* sp.-turtle grass pathosystem. *Mar. Ecol. Prog. Ser.* **581**, 119-133, doi:10.3354/meps12318 (2017).

12 Hasler-Sheetal, H., Castorani, M. C. N., Glud, R. N., Canfield, D. E. & Holmer, M. Metabolomics reveals cryptic interactive effects of species interactions and environmental stress on nitrogen and sulfur metabolism in seagrass. *Environmental Science & Technology* **50**, 11602-11609, doi:10.1021/acs.est.6b04647 (2016).

13 Villazan, B., Salo, T., Brun, F. G., Vergara, J. J. & Pedersen, M. F. High ammonium availability amplifies the adverse effect of low salinity on eelgrass *Zostera marina*. *Mar. Ecol. Prog. Ser.* **536**, 149-162, doi:10.3354/meps11435 (2015).

14 Salo, T. & Pedersen, M. F. Synergistic effects of altered salinity and temperature on estuarine eelgrass (*Zostera marina*) seedlings and clonal shoots. *J. Exp. Mar. Biol. Ecol.* **457**, 143-150, doi:10.1016/j.jembe.2014.04.008 (2014).

15 Kaldy, J. E. Effect of temperature and nutrient manipulations on eelgrass *Zostera marina* L. from the Pacific Northwest, USA. *J. Exp. Mar. Biol. Ecol.* **453**, 108-115, doi:10.1016/j.jembe.2013.12.020 (2014).

16 Govers, L. L. *et al.* Seagrasses are negatively affected by organic matter loading and *Arenicola marina* activity in a laboratory experiment. *Oecologia* **175**, 677-685, doi:10.1007/s00442-014-2916-8 (2014).

17 Fraser, M. W. *et al.* Extreme climate events lower resilience of foundation seagrass at edge of biogeographical range. *Journal of Ecology* **102**, 1528-1536, doi:10.1111/1365-2745.12300 (2014).

18 Villazan, B., Pedersen, M. F., Brun, F. G. & Vergara, J. J. Elevated ammonium concentrations and low light form a dangerous synergy for eelgrass *Zostera marina*. *Mar. Ecol. Prog. Ser.* **493**, 141-154, doi:10.3354/meps10517 (2013).

19 Gera, A., Pages, J. F., Romero, J. & Alcoverro, T. Combined effects of fragmentation and herbivory on *Posidonia oceanica* seagrass ecosystems. *Journal of Ecology* **101**, 1053-1061, doi:10.1111/1365-2745.12109 (2013).

20 Suykerbuyk, W. *et al.* Suppressing antagonistic bioengineering feedbacks doubles restoration success. *Ecological Applications* **22**, 1224-1231, doi:10.1890/11-1625.1 (2012).

21 Hoffle, H., Wernberg, T., Thomsen, M. S. & Holmer, M. Drift algae, an invasive snail and elevated temperature reduce ecological performance of a warm-temperate seagrass, through additive effects. *Mar. Ecol. Prog. Ser.* **450**, 67-85, doi:10.3354/meps09552 (2012).

22 Holmer, M., Wirachwong, P. & Thomsen, M. S. Negative effects of stress-resistant drift algae and high temperature on a small ephemeral seagrass species. *Mar. Biol.* **158**, 297-309, doi:10.1007/s00227-010-1559-5 (2011).

23 Blake, R. E. & Duffy, J. E. Grazer diversity affects resistance to multiple stressors in an experimental seagrass ecosystem. *Oikos* **119**, 1625-1635, doi:10.1111/j.1600-0706.2010.18419.x (2010).

24 Martinez-Luscher, J. & Holmer, M. Potential effects of the invasive species *Gracilaria vermiculophylla* on *Zostera marina* metabolism and survival. *Marine Environmental Research* **69**, 345-349, doi:10.1016/j.marenvres.2009.12.009 (2010).

25 Mascaro, O., Valdemarsen, T., Holmer, M., Perez, M. & Romero, J. Experimental manipulation of sediment organic content and water column aeration reduces *Zostera marina* (eelgrass) growth and survival. *J. Exp. Mar. Biol. Ecol.* **373**, 26-34, doi:10.1016/j.jembe.2009.03.001 (2009).

26 Jaschinski, S. & Sommer, U. Top-down and bottom-up control in an eelgrass-epiphyte system. *Oikos* **117**, 754-762, doi:10.1111/j.2008.0030-1299.16455.x (2008).

27 Brun, F. G. *et al.* Increased vulnerability of *Zostera noltii* to stress caused by low light and elevated ammonium levels under phosphate deficiency. *Mar. Ecol. Prog. Ser.* **365**, 67-75, doi:10.3354/meps07512 (2008).

28 Perez, M., Invers, O., Ruiz, J. M., Frederiksen, M. S. & Holmer, M. Physiological responses of the seagrass *Posidonia oceanica* to elevated organic matter content in sediments: An experimental assessment. *J. Exp. Mar. Biol. Ecol.* **344**, 149-160, doi:10.1016/j.jembe.2006.12.020 (2007).

29 Koch, M. S., Schopmeyer, S. A., Holmer, M., Madden, C. J. & Kyhn-Hansen, C. *Thalassia testudinum* response to the interactive stressors hypersalinity, sulfide and hypoxia. *Aquatic Bot.* **87**, 104-110, doi:10.1016/j.aquabot.2007.03.004 (2007).

30 Koch, M. S., Schopmeyer, S., Kyhn-Hansen, C. & Madden, C. J. Synergistic effects of high temperature and sulfide on tropical seagrass. *J. Exp. Mar. Biol. Ecol.* **341**, 91-101, doi:10.1016/j.jembe.2006.10.004 (2007).

31 Douglass, J. G., Duffy, J. E., Spivak, A. C. & Richardson, J. P. Nutrient versus consumer control of community structure in a Chesapeake Bay eelgrass habitat. *Mar. Ecol. Prog. Ser.* **348**, 71-83, doi:10.3354/meps07091 (2007).

32 Kahn, A. E. & Durako, M. J. *Thalassia testudinum* seedling responses to changes in salinity and nitrogen levels. *J. Exp. Mar. Biol. Ecol.* **335**, 1-12, doi:10.1016/j.jembe.2006.02.011 (2006).

33 Koch, M. S. & Erskine, J. M. Sulfide as a phytotoxin to the tropical seagrass *Thalassia testudinum*: interactions with light, salinity and temperature. *J. Exp. Mar. Biol. Ecol.* **266**, 81-95, doi:10.1016/s0022-0981(01)00339-2 (2001).

34 Goodman, J. L., Moore, K. A. & Dennison, W. C. Photosynthetic responses of eelgrass (*Zostera-marina* L) to light and sediment sulfide in a shallow barrier-island lagoon. *Aquatic Bot.* **50**, 37-47, doi:10.1016/0304-3770(94)00444-q (1995).
